# Deep Functional Profiling of Wild Animal Microbiomes Reveals Probiotic *Bacillus pumilus* Strains with a Common Biosynthetic Fingerprint

**DOI:** 10.3390/ijms23031168

**Published:** 2022-01-21

**Authors:** Margarita N. Baranova, Arsen M. Kudzhaev, Yuliana A. Mokrushina, Vladislav V. Babenko, Maria A. Kornienko, Maja V. Malakhova, Victor G. Yudin, Maria P. Rubtsova, Arthur Zalevsky, Olga A. Belozerova, Sergey Kovalchuk, Yuriy N. Zhuravlev, Elena N. Ilina, Alexander G. Gabibov, Ivan V. Smirnov, Stanislav S. Terekhov

**Affiliations:** 1 Shemyakin-Ovchinnikov Institute of Bioorganic Chemistry of the Russian Academy of Sciences, 117997 Moscow, Russia; baranova@ibch.ru (M.N.B.); kudzhaev_arsen@mail.ru (A.M.K.); yuliana256@mail.ru (Y.A.M.); aozalevsky@gmail.com (A.Z.); o.belozyorova@gmail.com (O.A.B.); xerx222@gmail.com (S.K.); 2 Department of Chemistry, Lomonosov Moscow State University, 119991 Moscow, Russia; mprubtsova@gmail.com; 3 Federal Research and Clinical Centre of Physical-Chemical Medicine of Federal Medical Biological Agency, 119435 Moscow, Russia; daniorerio34@gmail.com (V.V.B.); manja_k@list.ru (M.A.K.); maja_m@mail.ru (M.V.M.); ilinaen@gmail.com (E.N.I.); 4 Federal Scientific Center of the East Asia Terrestrial Biodiversity, Far-Eastern Branch of Russian Academy of Science, 690022 Vladivostok, Russia; vgyudin@rambler.ru (V.G.Y.); zhuravlev@biosoil.ru (Y.N.Z.)

**Keywords:** ultrahigh-throughput screening, biodiversity, wild animal microbiomes, probiotic discovery, droplet microfluidics, amicoumacin, biosynthetic gene clusters (BGCs), metabolomic fingerprinting

## Abstract

The biodiversity of microorganisms is maintained by intricate nets of interactions between competing species. Impaired functionality of human microbiomes correlates with their reduced biodiversity originating from aseptic environmental conditions and antibiotic use. Microbiomes of wild animals are free of these selective pressures. Microbiota provides a protecting shield from invasion by pathogens in the wild, outcompeting their growth in specific ecological niches. We applied ultrahigh-throughput microfluidic technologies for functional profiling of microbiomes of wild animals, including the skin beetle, Siberian lynx, common raccoon dog, and East Siberian brown bear. Single-cell screening of the most efficient killers of the common human pathogen *Staphylococcus aureus* resulted in repeated isolation of *Bacillus pumilus* strains. While isolated strains had different phenotypes, all of them displayed a similar set of biosynthetic gene clusters (BGCs) encoding antibiotic amicoumacin, siderophore bacillibactin, and putative analogs of antimicrobials including bacilysin, surfactin, desferrioxamine, and class IId cyclical bacteriocin. Amicoumacin A (Ami) was identified as a major antibacterial metabolite of these strains mediating their antagonistic activity. Genome mining indicates that Ami BGCs with this architecture subdivide into three distinct families, characteristic of the *B. pumilus*, *B. subtilis*, and *Paenibacillus* species. While Ami itself displays mediocre activity against the majority of Gram-negative bacteria, isolated *B. pumilus* strains efficiently inhibit the growth of both Gram-positive *S. aureus* and Gram-negative *E. coli* in coculture. We believe that the expanded antagonistic activity spectrum of Ami-producing *B. pumilus* can be attributed to the metabolomic profile predetermined by their biosynthetic fingerprint. Ultrahigh-throughput isolation of natural probiotic strains from wild animal microbiomes, as well as their metabolic reprogramming, opens up a new avenue for pathogen control and microbiome remodeling in the food industry, agriculture, and healthcare.

## 1. Introduction

The biodiversity of microbial communities is maintained by the counteraction of the production and degradation of antibiotics [1]. The external influence of disinfectants and antibiotics drives additional selective pressures, dramatically reducing the biodiversity of microbiomes [2,3] and providing conditions for antibiotic resistance propagation [4] and evolution [5]. Reduced microbiome biodiversity, in turn, is attributed to its impaired functionality [6,7] and health disorders [8], including the emergence and transmission of infectious diseases [9], autoimmune diseases [10,11], and allergic diseases [12,13]. Hence, directed microbiome remodeling provides pronounced health benefits and creates alternative therapeutic strategies [14]. Probiotics are applied to restore the composition of the microbiome and introduce beneficial functions to microbial communities [15]. Commensals serve as probiotics competing with opportunistic pathogens in natural microbiomes [16]. Therefore, probiotic strains may be isolated in microbiomes resistant to pathogen invasion [17]. Looking for diverse naïve microbiomes as sources for probiotic strains, we addressed wild animals living in natural aseptic conditions and facing a broad range of microorganisms, including pathogens and invasive species.

To explore probiotic strains in wild animal microbiomes, we applied an ultrahigh-throughput microfluidic platform for profiling of antimicrobial activity on a single-cell level [18,19,20]. This technology enables us to select the most efficient bacterial antagonists from the whole microbiome. A critical step is a single-cell cocultivation of microbiome biodiversity together with a reporter fluorescent pathogen in emulsion droplets, followed by the selection of reporter-free droplets using fluorescence-activated cell sorting and regeneration of effector strains. The principal advantage of this platform is its unprecedented productivity, which allows us to uncover rare probiotic strains with the most pronounced pathogen killing. Previously, the highly potent *Bacillus pumilus* 124 strain producing antibiotic amicoumacin A (Ami) was identified as a component of the microbiome of the East Siberian brown bear (*Ursus arctos collaris*), which is highly active against the common human pathogen *Staphylococcus aureus* [19]. Originally, Ami was isolated from the *B. pumilus* BN-103 strain [21]. However, numerous *B. subtilis* selected for their antibacterial activity also mediate their antagonistic properties via Ami production, and some of them were isolated from the human microbiome [22,23]. Both *B. subtilis* and *B. pumilus* strains are widely used as probiotics for broiler chickens [24], pigs [25], aquaculture [26], and human use [27]. Despite their broad use, their genotypes and biosynthetic profiles are rarely characterized in detail.

The ultrahigh-throughput microfluidic platform revealed different probiotic *B. pumilus* strains from microbiomes of diverse wild animals, including the skin beetle (*Dermestes maculatus*), Siberian lynx (*Lynx lynx wrangeli*), common raccoon dog (*Nyctereutes procyonoides*), and previously reported East Siberian brown bear (*Ursus arctos collaris*). These strains were selected based on their anti-*S. aureus* activity and demonstrated prominent antagonistic properties toward different bacteria. Ami was determined as the main antibiotic component produced by the selected strains. While Ami is active primarily against Gram-positive bacteria [20], antagonistic properties of isolates were also observed against some Gram-negatives. Whole-genome sequencing and genome mining enabled us to depict a biosynthetic fingerprint of isolated *B. pumilus* strains. All their genomes have a similar set of biosynthetic gene clusters (BGCs) encoding Ami, siderophore bacillibactin, and putative analogs of antimicrobials including bacilysin, surfactin, desferrioxamine, and class IId cyclical bacteriocin. We speculate that this genotype mediates the prominent antagonism observed in coculture experiments. Genome mining indicates that Ami BGCs subdivide into three distinct families characteristic of the (i) *B. pumilus* type, (ii) *B. subtilis* type, and (iii) *Paenibacillus* type. Hence, similar antagonistic properties may be observed among these species.

We believe that the detailed genomic description and analysis of BGCs provide an essential genotype-phenotype link that will further our understanding of the exact impact of probiotics on pathogens and hosts. Ultrahigh-throughput technologies facilitate the isolation of natural probiotic strains from various microbiomes. A deep understanding of their genotype is essential for the metabolic reprogramming of probiotics. Designer probiotics for pathogen control and microbiome remodeling will provide direct benefits in the food industry, agriculture, and healthcare.

## 2. Results

### 2.1. Deep Functional Profiling of Microbiomes of Wild Animals

**Figure 1:**
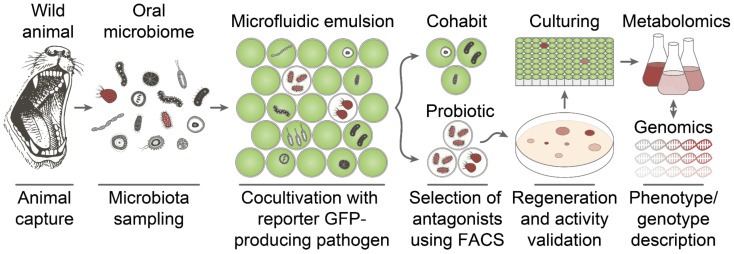
Principal scheme of ultrahigh-throughput selection of probiotics. The wild animal is captured and the oral microbiome is isolated by non-invasive probing. Single cells of the microbiome community are coencapsulated with a reporter GFP-producing pathogen in biocompatible droplets of a microfluidic double water-in-oil-in-water emulsion. Cocultivation of a bacterial community with reporter bacteria in droplets results in two distinct populations containing bacterial cohabits and probiotic bacteria, thus mediating pathogen killing. The latter is selected by a low level of GFP fluorescence using FACS. The selected droplets are plated on agar to regenerate culturable probiotic strains. Regenerated clones are validated by coculturing assays and analyzed by activity-guided metabolomics and genomics. Detailed phenotype and genotype descriptions enable us to identify antibiotics and their biosynthetic gene clusters.

Previously, an ultrahigh-throughput microfluidic platform was applied to select the most active bacterial antagonists from the human microbiome [18]. In this study, this technology was adopted to select bacterial antagonists from the microbiomes of different wild animals. It was assumed that living in the wild provides additional selective pressure on wild animal microbiomes, promoting them to serve as first-line gates for pathogen invasion. Since wound licking does not result in regular acute sepsis events, it was suggested that animal bacterial communities may contain probiotic bacteria protecting hosts from pathogens. Microbiomes of wild animals, including the skin beetle (*Dermestes maculatus*), Siberian lynx (*Lynx lynx wrangeli*), common raccoon dog (*Nyctereutes procyonoides*), and East Siberian brown bear (*Ursus arctos collaris*) were investigated to select the most active probiotic strains using an ultrahigh-throughput microfluidic platform (Figure 1).

*Staphylococcus aureus* was used as a model pathogen to select bacterial killers, abolishing its growth in coculture. Single-cell cocultivation of bacteria from microbiomes of wild animals with the reporter *S. aureus* strain in microfluidic droplets was followed by isolation of *S. aureus* killers by fluorescence-activated cell sorting (FACS) of droplet microcompartments occupied by *S. aureus* antagonists. The selected bacteria were regenerated from droplets and screened for anti-*S. aureus* activity, and the most efficient bacterial antagonists were collected for further characterization by genomics and metabolomics. Repeated isolation of *B. pumilus* strains with prominent *S. aureus* antagonism was observed using this platform. *B. pumilus* represented ~0.5–2% of the culturable component of wild animal microbiomes.

### 2.2. Biosynthetic Fingerprint of Isolated B. pumilus Strains

**Figure 2:**
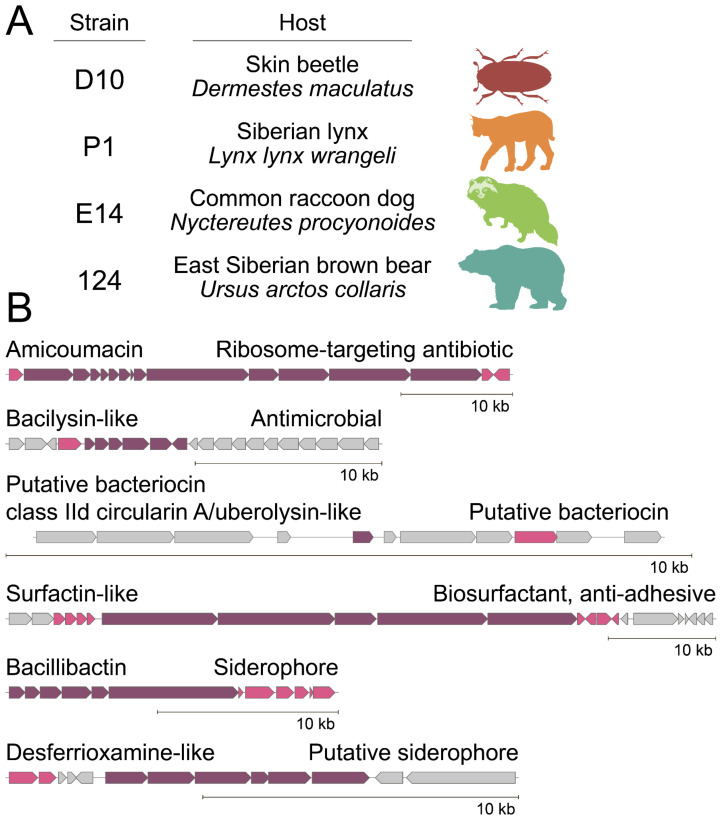
Representative *B. pumilus* strains isolated from different wild hosts. (**A**) Summary of their origin and (**B**) a common set of BGCs encoding Ami, siderophore bacillibactin, and putative analogs of antimicrobials including bacilysin, class IId cyclical bacteriocin, surfactin, and desferrioxamine. Core BGC proteins and related genes (transporters, resistance, and modifying enzymes) are colored with violet and pink, respectively. Scale bar: 10 kb.

Isolated *B. pumilus* strains demonstrated different phenotypes (colony morphology and mobility) that may be associated with their different ecological microenvironments in their hosts. Whole-genome sequencing was applied to understand the biosynthetic potential of representative *B. pumilus* strains (Figure 2A).

Despite different phenotypes observed and specific BGCs detected, e.g., lantibiotics in *B. pumilus* P1 strain, genome mining revealed a common set of BGCs characteristic of antagonistic *B. pumilus* strains (Figure 2B). BGCs of the antibiotic Ami [19,22] and siderophore bacillibactin [28] were detected in their genomes. Moreover, a number of BGCs encoding putative analogs of antimicrobials, including bacilysin [29], class IId cyclical bacteriocin, surfactin [30], and desferrioxamine, were detected.

### 2.3. Antagonistic Properties of B. pumilus Strains

**Figure 3:**
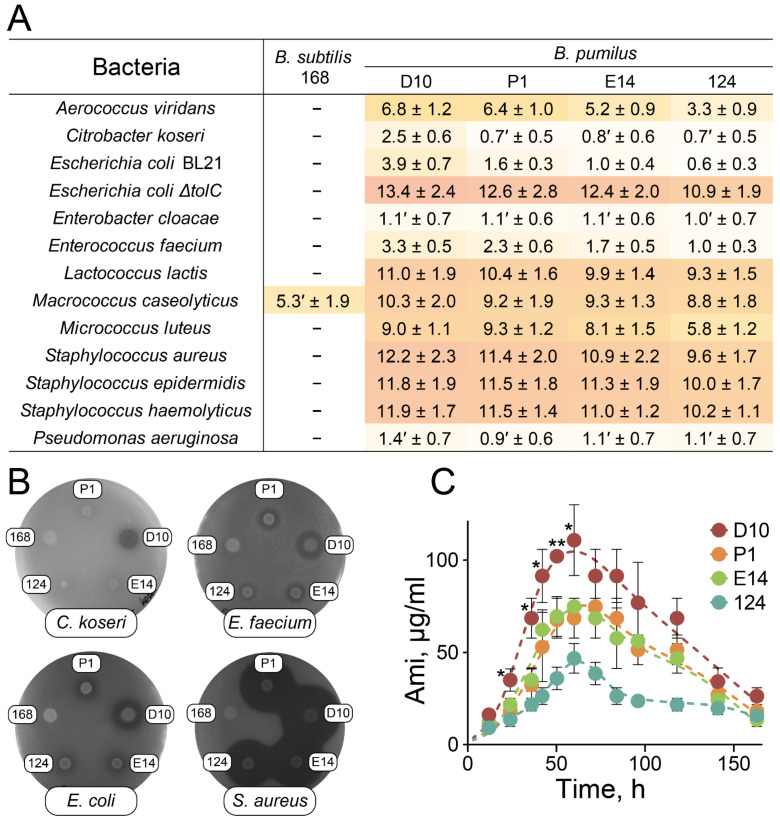
Antagonistic properties of *B. pumilus* strains and Ami production. (**A**) Diameters of clearance zones (mm) of selected *B. pumilus* strains (D10, P1, E14, and 124) were observed for various target pathogens using agar overlay assay. *B. subtilis* 168 was used as a negative control. The (′) symbol corresponds to diffusive clearance zones. Clearance zones were not detected for *Acinetobacter baumanii*, *Klebsiella pneumoniae*, *Morganella morganii*, *Salmonella enterica*, and *Serratia marcenscens*. Heatmap indicates the diameter value. Data represent the mean of three biological replicates ± SD. (**B**) Representative agar plates with colonies of probiotic *B. pumilus* strains overlaid with *C. koseri*, *E. faecium*, *E. coli* BL21, and *S. aureus*. (**C**) Dynamics of Ami production by selected *B. pumilus* strains. Ami concentration (dots) was determined by HPLC and antibacterial activity assay of culture media in triplicate. * *p* < 0.05; ** *p* < 0.01. Data represent mean ± SD.

An agar overlay assay was performed to estimate the activity spectrum of selected probiotic strains (Figure 3). *B. subtilis* 168 was used as a negative control since it has impaired secondary metabolite biosynthesis resulting from inactive 4-phosphopantetheinyl transferase [31].

All the selected strains efficiently inhibited the growth of Gram-positive non-spore-forming bacteria, displaying mediocre activity against Gram-negative strains (Figure 3A,B). Large clearance zones were observed for various *Staphylococcus* species (*S. aureus*, *S. epidermidis,* and *S. haemolyticus*), *Lactococcus lactis*, *Macrococcus caseolyticus*, *Micrococcus luteus*, *Aerococcus viridans*, and *Escherichia coli ΔtolC*. A less pronounced inhibitory effect was detected for *Enterococcus faecium*, *E. coli* BL21, and *Citrobacter koseri*. Small diffusive zones of growth inhibition were observed for *Pseudomonas aeruginosa* and *Enterobacter cloacae*. No clearance zones were detected for *Acinetobacter baumanii*, *Klebsiella pneumoniae*, *Morganella morganii*, *Salmonella enterica*, and *Serratia marcenscens*. *B. pumilus* D10 demonstrated the most prominent antagonistic activity on agar plates (Figure 3A,B).

The antagonistic properties of *B. pumilus* strains generally correlated with the Ami activity spectrum reported previously [20]. Activity-based metabolomic analysis revealed that Ami was the only antibiotic component mediating the bactericidal effect against *S. aureus* in culture media of *B. pumilus* D10, P1, E14, and 124. Amicoumacin B (AmiB) and amicoumacin C (AmiC) resulting from Ami lactonization (AmiC) followed by hydrolysis (AmiB) were also detected as inactive metabolites of Ami (Figures S1–S4). The selected strains were compared based on their level of Ami production (Figure 3C). *B. pumilus* strains showed maximum Ami production after 60 h of cultivation. *B. pumilus* D10 produced the highest concentrations of Ami.

**Figure 4:**
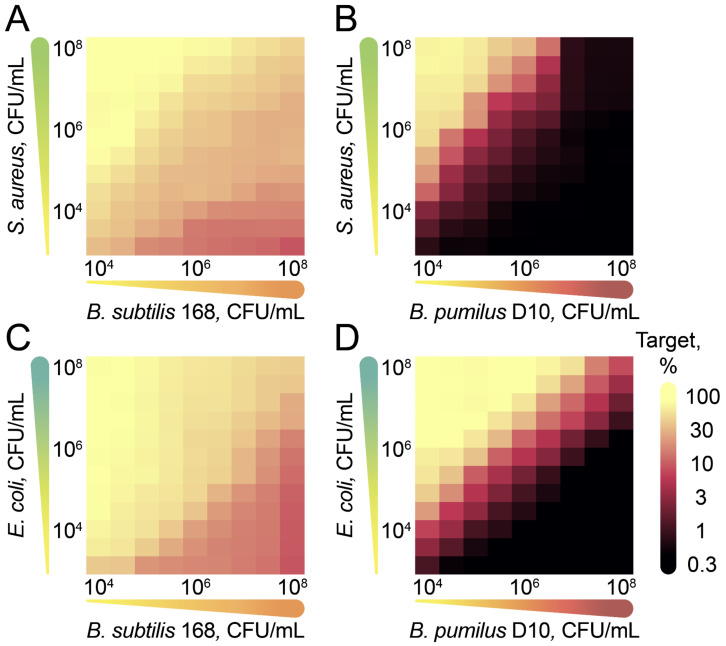
Antimicrobial activity landscapes of *B. pumilus* D10. Reporter *S. aureus* (**A**,**B**) and *E. coli* (**C**,**D**) were cocultivated with effector strains using various cell ratios. Effector *B. subtilis* 168 strain was used as a negative control. Target was analyzed by fluorescence of culture after 24 h of cocultivation. Heatmap indicates maximal target proliferation estimated by relative fluorescence level.

Cocultivation of the probiotic *B. pumilus* D10 strain with model reporter pathogens *S. aureus* and *E. coli* was provided using different target: effector ratios to detail its antagonistic landscape (Figure 4 and Figure S5). Reporter strains producing fluorescent proteins enabled time-resolved detecting of growth inhibition.

A fluorescence level above 20% of the maximal was observed for the control *B. subtilis* 168 even at low target: effector ratios (Figure 4A,C). Similar effects were observed for coculturing of *S. aureus* and *E. coli* cells (Figure S5). We associate this effect with the competition of microorganisms for nutrients. Unlike *B. subtilis* 168, *B. pumilus* D10 efficiently inhibited the growth of reporter bacteria (Figure 4B,D). The effect of cocultivation on the target bacteria was more complex than the effect of pure Ami. While it is impossible to eradicate *E. coli* cells using Ami alone, as little as 10^4^ CFU/mL *B. pumilus* D10 provided detectable eradication of both *S. aureus* and *E. coli* within the first 24 h. The outstanding efficacy of the probiotic *B. pumilus* in coculture is essential for their ecology. Apparently, it is associated with the additional biosynthetic arsenal reported previously. We suggest that the additional secondary metabolites either potentiate Ami or increase the permeability of target cells toward Ami.

### 2.4. Biodiversity of Ami Clusters

**Figure 5:**
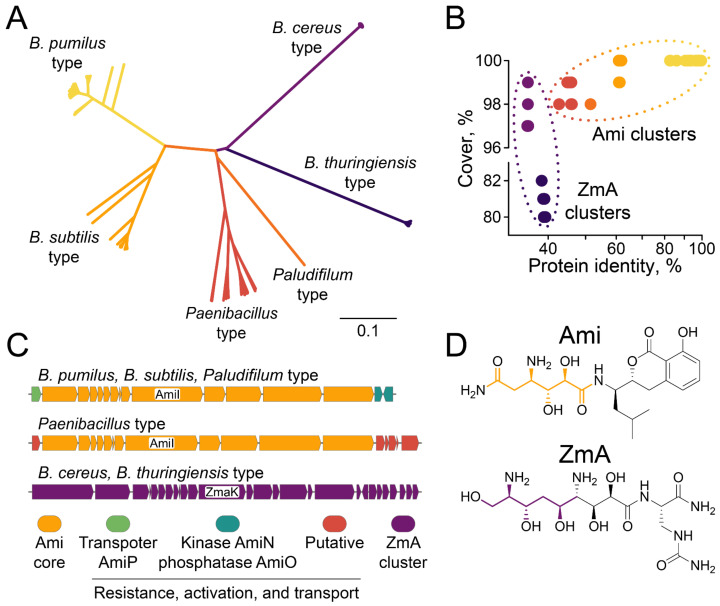
Biodiversity of Ami clusters. (**A**) A phylogenetic tree of Ami clusters was built based on the key hybrid PKS/NRPS enzyme AmiI homology. BGCs encoding Ami (*B. pumilus* type, *B. subtilis* type, *Paludifilum* type, and *Paenibacillus* type) are colored with warm colors (yellow, orange, and red). BGCs encoding zwittermicin A (ZmA) are colored with cold colors (sapphire and violet for *B. thuringiensis* type and *B. cereus* type, respectively). (**B**) Protein identity and protein cover of AmiI homologs. Ami cluster family and ZmA cluster family are highlighted with a dotted line. (**C**) Comparative analysis of the architecture of Ami clusters and ZmA clusters and (**D**) their biosynthetic products. Core Ami enzymes are colored with orange. ZmA cluster is colored with violet. AmiI in Ami cluster and its homolog ZmaK in ZmA cluster are subscribed and their biosynthetic fragments are highlighted with orange and violet in Ami and ZmA structures, respectively. Transporter AmiP is colored with green. Kinase AmiN and phosphatase AmiO mediate self-resistance toward Ami and Ami activation, respectively. AmiN and AmiO are colored with aquamarine. *Paenibacillus* genes encoding proteins putatively associated with transport/self-resistance are colored with red.

The results obtained indicate a high impact of Ami-producing strains on bacterial communities. Genome mining of Ami clusters provided a detailed description of their natural landscape (Figure 5).

Generally, Ami clusters were identified in numerous *Bacilli* and could be subdivided into three main families characteristic of (i) *B. pumilus* type (*B. pumilus, B. altitudinis, B. safensis, B. stratosphericus, B. zhangzhouensis, Priestia endophytica)*, (ii) *B. subtilis* type (*B. subtilis, B. bingmayongensis, B. rugosus, B. atrophaeus, B. inaquosorum, B. vallismortis*, *B. swezeyi*), and (iii) *Paenibacillus* type (*P. dendritiformis, P. solani, P. lautus, P. apiaries, P. thiaminolyticus, P. bouchesdurhonensis, P. lentus*) (Figure 5A and Figure S6). An Ami cluster was also identified in the rare genome of *Paludifilum halophilum*, indicating that Ami production may be observed for some *Thermoactinomycetaceae*. A closely related BGC of antibiotic zwittermicin A (ZmA) was frequently identified in *B. cereus* and *B. thuringiensis* genomes. The key hybrid PKS/NRPS enzyme AmiI encoded the structural fragment that differed between Ami and ZmA (Figure 5D). That enabled distinguishing between Ami clusters and related ZmA clusters (Figure 5B,C). ZmaK is an AmiI homolog in the ZmA cluster with a protein identity < 40%. Identical chemical structures were previously reported for *B. pumilus* and *B. subtilis*-type clusters, despite them having a protein identity of about 60% [19,22]. Hence, we propose a similar structure for products of *Paenibacillus*-type Ami-like BGCs with a synonymous modular organization (Figure 5C).

Ami transport and self-resistance mechanisms differ between *Bacillus* and *Paenibacillus*. Kinase AmiN and phosphatase AmiO are located downstream of the Ami cluster core, mediating Ami inactivation and reactivation, respectively [19,32]. Kinase AmiN is conservative in *B. pumilus*, *B. subtilis*, and *Paludifilum*-type clusters going in the same orientation as core Ami cluster genes (Figure S6). Phosphatase AmiO is not essential for Ami clusters in *Priestia endophytica* and *Paludifilum halophilum*, indicating that alternative phosphatases in genomes of these bacteria are involved in Ami dephosphorylation. AmiO has the opposite orientation in the majority of *B. pumilus* and *B. subtilis*-type clusters, while direct orientation and transposon flanking were observed in a minor population of distant clusters (Figure S2). That may indicate the evolution of cluster regulation and compactization. Homologs of kinase AmiN and phosphatase AmiO were not detected in *Paenibacillus*-type Ami-like BGCs. Instead, putative methyltransferase and acetyltransferase were detected downstream of the core Ami cluster genes (Figure 5C and Figure S6). We suggest their involvement in self-resistance since it was reported that acetyltransferases mediate self-resistance in Ami-producing *Xenorhabdus bovienii* [33] and a related ZmA cluster [34]. Similarly, a transporter AmiP located upstream of core genes of the Ami cluster was not detected in *Paenibacillus*-type Ami-like BGCs (Figure 5C and Figure S6). Instead, a putative C39 peptidase-containing protein was conservatively found upstream of the Ami cluster core in *Paenibacillus*-type Ami-like BGCs. We propose its involvement in transport/deacetylation in *Paenibacillus*-type BGCs.

## 3. Discussion

Antibiotic misuse is particularly dangerous, and not only because it provides selective pressure for the evolution of antibiotic resistance (AR). Even more importantly, antibiotics clear the natural protective shield provided by commensals, thus outcompeting pathogens in their microenvironments. The development of probiotic strains is important in this context since they may act in a dual mode: (i) direct killing AR pathogens, and (ii) reconstituting the degraded microbiota shield. Moreover, probiotics may provide essential signals for the host immunity, regulating autoimmunity and providing stimuli often found in nature but lost during urbanization.

Here, ultrahigh-throughput microfluidic technology was applied for the isolation of bacterial killers serving as natural probiotics in the microbiomes of different wild animals. Despite the large differences in microbiota composition and the presence of source-specific killing strains, antagonistic strains of *B. pumilus* were isolated repeatedly. While a more extensive study should reveal the prevalence of the described *B. pumilus* strains in wild animals and depict their impact on hosts, the repeated isolation of antagonistic *B. pumilus* strains indicates their ubiquity and significant influence on ecological niches. Metabolomics and genomics were applied to provide a comprehensive description of these strains. Surprisingly, a common set of BGCs encoding Ami, siderophore bacillibactin, and putative analogs of antimicrobials including bacilysin, class IId cyclical bacteriocin, surfactin, and desferrioxamine was detected. Activity-based metabolomic analysis revealed that Ami is the principal antibiotic component produced by *B. pumilus*. Ami is a potent inhibitor of translation [35] displaying mediocre activity against the majority of Gram-negative bacteria [20]. Isolated *B. pumilus* strains efficiently inhibited the growth of both Gram-positive *S. aureus* and Gram-negative *E. coli* in coculture. We suggest that probiotic *B. pumilus* strains display their antagonistic properties by the complex action of *B. pumilus* secondary metabolites. While the exact metabolite(s), potentiating Ami are still to be determined, we believe that the discovered biosynthetic fingerprint is essential for the described antagonism.

Genome mining indicates that Ami-producing strains may be observed in numerous *Bacillus*, *Paenibacillus*, and related species. The core Ami cluster genes have similar architectures for *Bacillus* and *Paenibacillus.* However, mechanisms of Ami transport and self-resistance discovered for Ami [19,32] are different in *Paenibacillus*. We speculate that Ami acetylation may take place, as was previously described in Ami-producing *Xenorhabdus bovienii* [33] and a related ZmA cluster [34]. The fact that Ami BGCs were identified both in Gram-negative *X. bovienii* [33] and Gram-positive *Bacillus* [19,22] is particularly interesting. These clusters have different architectures and operate different self-resistance mechanisms. However, they encode the same low-molecular metabolite, representing an outstanding example of the convergent evolution of BGCs. The described probiotic activity of isolated *B. pumilus* strains together with the interspecial diversity of Ami clusters imply a high impact of Ami-producing strains on bacterial communities.

The development of functional probiotics demands thorough characterization of their genotype and metabolomic profile. *Bacillus* species are common commercial probiotic products. However, particular probiotic strains may carry a latent threat in their genomes. That was reported for some *B. cereus* strains producing enterotoxins, which makes them unsafe for human use [27]. While homologs of Hbl and Nhe enterotoxins were not identified in the selected probiotic *B. pumilus* strains, their safety and impact on microbiomes are still to be determined precisely. The creation of designer probiotics with deleted toxins and transferred BGCs encoding microbiome reprogramming metabolites represents a new paradigm of probiotic development. We believe that ultrahigh-throughput isolation of natural probiotic strains from wildlife microbiomes, as well as their metabolic reprogramming, opens up a new avenue for pathogen control and microbiome remodeling in the food industry, agriculture, and healthcare.

## 4. Conclusions

Ultrahigh-throughput microfluidic technology was applied for the isolation of bacterial killers serving as natural probiotics in the microbiomes of different wild animals. Despite the large differences in microbiota composition and the presence of source-specific killing strains, antagonistic strains of *B. pumilus* were isolated repeatedly. Metabolomics and genomics were applied to provide a comprehensive description of these strains. A common biosynthetic fingerprint was detected. Activity-based metabolomic analysis revealed that Ami is the principal antibiotic component produced by *B. pumilus*. While Ami itself displays mediocre activity against Gram-negative bacteria, isolated *B. pumilus* strains efficiently inhibit the growth of both Gram-positive *S. aureus* and Gram-negative *E. coli* in coculture. We believe that the expanded antagonistic activity spectrum of Ami-producing *B. pumilus* can be attributed to the metabolomic profile predetermined by their biosynthetic fingerprint. Ultrahigh-throughput isolation of natural probiotic strains from wild animal microbiomes, as well as their metabolic reprogramming, opens up a new avenue for pathogen control and microbiome remodeling in the food industry, agriculture, and healthcare.

## 5. Materials and Methods

**Bacterial strains.** A bacterial collection of clinical isolates including *Acinetobacter baumannii, Aerococcus viridans, Citrobacter koseri, Enterobacter cloacae, Enterococcus faecium, Klebsiella pneumoniae, Lactococcus lactis, Macrococcus caseolyticus, Micrococcus luteus, Morganella morganii, Pseudomonas aeruginosa, Salmonella enterica, Staphylococcus epidermidis*, and *Staphylococcus haemolyticus* was kindly provided by Lytech Co. Ltd. (Moscow, Russia). GFP-producing *Staphylococcus aureus* was described previously [18]. *Escherichia coli* BW25113 ΔtolC was kindly provided by Ilya A. Osterman, Moscow State University. The *Bacillus subtilis* 168 ATCC 23857 *E. coli* BL21 (DE3) strain (Invitrogen, Waltham, MA, USA) was transformed with a pKatushka2S-B vector (Evrogen, Moscow, Russia) to provide far-red fluorescent reporter *E. coli* cells.

**Microbiota collection.** Oral microbiota samples were collected from healthy a Siberian lynx (*Lynx lynx wrangeli*) and common raccoon dog (*Nyctereutes procyonoides*) using a noninvasive probe immediately after capture in Primorsky Krai, Russia. The noninvasive probe containing the collected microbiota samples was thoroughly washed with a sterile medium for microbiota cryopreservation. The cell suspension was immediately frozen in liquid nitrogen, transported in dry ice, and stored in liquid nitrogen. The oral microbiota of an East Siberian brown bear (*Ursus arctos collaris*) was collected previously [19] and stored in liquid nitrogen. Skin beetle (*Dermestes maculatus*) imago were extensively washed with a sterile PBS buffer and homogenized using a manual French press. The residual was thoroughly washed with a sterile medium, frozen, and stored in liquid nitrogen.

**Deep functional profiling.** The application of ultrahigh-throughput microfluidic technology for the selection of bacteria displaying anti-*S. aureus* activity was described in detail previously [18,19]. Target *S. aureus* cells producing a GFP reporter were vitally stained with sulfo-Cyanine5 NHS (Lumiprobe, Moscow, Russia), washed, filtered using 20 μm solvent filters (A-313, IDEX, Northbrook, IL, USA), and coencapsulated with a microbiota suspension in droplets of microfluidic double emulsion (MDE), using 20 μm microfluidic chips produced via soft lithography. The microbiota samples were unfrozen directly before encapsulation, resuspended in a BHI broth (BD, Franklin Lakes, NJ, USA), and filtered through 40 μm cell strainers (Greiner Bio-One, Kremsmünster, Austria). After overnight incubation at 30–35 °C, Calcein Violet AM (Thermo Fisher Scientific, Waltham, MA, USA) was added to the droplet emulsion to the final concentration of 10 μM. Subsequently, the droplets with simultaneous sCy5^high^, GFP^low^, and Calcein Violet^high^ fluorescence were sorted using a FACSAria III cell sorter (BD, USA). Bacterial colonies were regenerated after plating on BHI–agar (BD, Franklin Lakes, NJ, USA) and tested for anti-*S. aureus* activity using the agar overlay assay. Bacterial clones demonstrating *S. aureus* antagonism were identified by mass spectrometry and studied by metabolomic analysis and whole-genome sequencing.

**Identification of bacteria using mass spectrometry.** Bacterial cells were spotted on a sample spot of a MALDI target plate (MSP 96 target, ground steel; Bruker Daltonics, Billerica, MA, USA) and were overlaid with HCCA matrix solution (saturated solution of α-4-cyano-hydroxycinnamic acid; Bruker Daltonics, Billerica, MA, USA) in 50% acetonitrile (Sigma-Aldrich, St. Louis, MO, USA) and 2.5% trifluoroacetic acid solution (Sigma-Aldrich, St. Louis, MO, USA). Mass spectra profiles were acquired using a Microflex spectrometer (Bruker Daltonics, Billerica, MA, USA). The molecular ions were measured automatically in the linear positive ion mode with the instrument parameters optimized for a range of 2000–20,000 *m*/*z*. The software packages flexControl 3.0 (Bruker Daltonics, Billerica, MA, USA) and flexAnalysis 3.0 (Bruker Daltonics, Billerica, MA, USA) were used for mass spectra recording and processing. Spectra identification and analysis were carried out using the MALDI Biotyper 3.0 (Bruker Daltonics, Billerica, MA, USA). The identification was performed by comparing the obtained spectra with those in the MALDI Biotyper 3.0 library (version 3.2.1.1).

**Whole-genome sequencing and bioinformatic analysis.** Pure genomic DNA (approx. 500 ng) was fragmented to a mean size of 200–300 bp using the Covaris S220 System (Covaris, Woburn, MA, USA). A KAPA Library Preparation Kit (KAPA Biosystems, Wilmington, MA, USA) was employed to prepare a barcoded shotgun library. Emulsion PCR was performed using a One Touch system (ThermoFisher Scientific, Waltham, MA, USA). Beads were prepared using the One Touch 2 and Template Kit v2, and sequencing was performed using an Ion Proton 200 Sequencing Kit v2 and P1 Ion chip. De novo assembly was performed by SPAdes 3.14.0 [36] using default parameters. Identification of the protein-coding sequences and primary annotation were performed using PROKKA v1.14.6 [37]. Identification of biosynthetic gene clusters and NRPS modular organization was performed with antiSMASH 6.0 [38]. Comparative analysis of homologous gene clusters was provided by MultiGeneBlast [39].

**Agar overlay assay.** Antagonistic *Bacillus* strains were grown overnight in liquid cultures at 30 °C. Taken from those cultures, 2 µL aliquots were plated on 2YT (BD, Franklin Lakes, NJ, USA) agar plates to form regular colonies. The colonies were cultivated at 30 °C followed by chloroform vapor treatment for 5 min. The agar overlay assay was performed using soft agar (8 g/L tryptone, 5 g/L yeast extract, 2.5 g/L NaCl, 0.5% agar). Soft agar was melted, cooled to 40 °C, and inoculated with an overnight culture of the target microorganism using a 1:1000 dilution. Next, 7 mL of resulting liquid soft agar was used to overlay *Bacillus* colonies. Agar plates were incubated overnight at 30 °C after soft-agar solidification. Clearance zones were measured in three biological replicates.

**Metabolomic analysis.** *B. pumilus* D10, P1, E14, and 124 culture media collected after 60 h of cultivation were used to determine the major active compound for each strain. Cells were pelleted at 10,000× *g*, the supernatant was filtered using a Millistak + HC Pod Depth Filter (Millipore, Billerica, MA, USA), and then it was fractionated with a Symmetry C18 (Waters) RP-HPLC column using buffer A (20 mM NH_4_OAc pH 5.0, 5% ACN) and B (20 mM NH_4_OAc pH 5.0, 80% ACN), with a flow rate of 1 mL/min and 40-min linear gradient. Then, 1-min fractions were collected and analyzed for antibiotic activity using reporter *S. aureus* and *E. coli* cells. Antibiotic activity was detected only in fractions corresponding to Ami. LC-MS analysis was carried out on an Ultimate 3000 RSLCnano HPLC system connected to an Orbitrap Fusion Lumos mass spectrometer (ThermoFisher Scientific, Waltham, MA, USA), with the loading pump used for analytical flow-gradient delivery. Samples were separated on a Luna Omega C18 1.6 µm 100 Å column 100 × 2.1 mm at a 200 µL/min flow rate. Separation was done by a gradient of 99.9% ACN, 10 mM ammonium formate, and 0.1% FA (Buffer B), or 99.9% H_2_O, 10 mM ammonium formate, and 0.1% FA (Buffer A): 5% B at 0 min, 5% B at 5 min, and 99% B at 20 min, followed by a 5 min wash at 99% B and 10 min equilibration at 5% B before the next run. UV data were collected at 260 and 315 nm. MS1 spectra were collected in the positive ion mode at a 30 K Orbitrap resolution and in the profile mode with a 200–2000 a.e.m mass range and RF lens 30%. For the rest of the MS1 parameters, as well as for the ESI parameters, the default values suggested by Xcalibur software ver. 4.3.73.11 (ThermoFisher Scientific, Waltham, MA, USA) were taken. MS2 precursors were selected based on the MS1 intensity: the intensity threshold was 5 × 104 with the dynamic exclusion set to 10 s after two selections, with the mass tolerance of 10 ppm and isotope exclusion. MS2 spectra were collected at 15-K resolution in the centroid mode. The isolation window was set to 1/6 *m*/*z* with no offset and the quadrupole isolation mode. Fragmentation was done by HCD with a stepped CE of 20, 35, and 50%. The rest of the MS2 parameters were taken as default values. The total MS1-MS2 cycle time was set to 1 s. Ami and its metabolites were identified with molecular masses and their fragmentation spectra were as described previously [19].

**Antibiotic activity testing.** Inhibition of bacterial cell growth was measured by a doubling dilution of culture media and C18 HPLC fractions in a 2YT medium supplemented with 20 µg/mL chloramphenicol inoculated with *E. coli* or *S. aureus* to OD_600_ ~0.001. After overnight incubation at 30 °C, bacterial growth was analyzed by GFP fluorescence (λ_ex_/λ_em_ = 488/513 nm) and OD_600_ using a Varioskan Flash Multimode Reader (ThermoFisher Scientific, Waltham, MA, USA).

**Quantification of Ami production.***B. pumilus* strains were cultivated in SYC medium (40 g/L sucrose, 5 g/L yeast extract, 4 g/L CaCO_3_, 1.5 g/L K_2_HPO_4_, 2 g/L glucose, 2 g/L NaCl, 1.5 g/L MgSO_4_, 2 g/L (NH_4_)_2_SO_4_, 0.01 g/L FeSO_4_, 0.01 g/L MnCl_2_) at 30 °C. *B. pumilus* strains were inoculated from an overnight culture (using 1:100 dilution) and cultivated using 750-mL flasks in 100 mL with 220 rpm shaking. Culture media were analyzed by RP-HPLC as was previously described. Amicoumacin and its derivatives were monitored by absorbance at 315 nm. The concentration of Ami was measured using the purified Ami standard, $\varepsilon_{315\;\mathrm{nm}}^{\mathrm{MeOH}}=4380\;M^{-1}{\mathrm{cm}}^{-1}$.

**Growth inhibition landscapes.** The inhibitory landscapes were obtained by cocultivation of reporter cells with antagonistic *B. pumilus* strains using various cell ratios. Target reporter bacteria *S. aureus* and *E. coli* produced intracellular GFP and Katushka2S fluorescent proteins, respectively. *B. subtilis* 168 was used as a negative control. Concentrations of target and effector bacteria were varied in the 10^3^–10^8^ CFU/mL range and 10^4^–10^8^ CFU/mL range, respectively. Serial three-fold dilutions in a 2YT medium were used. Cocultures were cultivated in 96-well plates using a microplate shaker at 35 °C and 600 rpm for 24 h. The target bacteria growth was monitored by GFP and Katushka2S fluorescence using a Varioskan Flash multimode reader (Thermo Fisher Scientific, Waltham, MA, USA).

## Data Availability

The whole genome sequencing data of *B. pumilus* strain 124 from the East Siberian brown bear is available in the GenBank database, https://www.ncbi.nlm.nih.gov/genbank/ (accession no. QENN00000000) (accessed on 15 November 2021). A representative Ami BGC from *B. pumilus* P1 isolated from the Siberian lynx was deposited in the GenBank database, https://www.ncbi.nlm.nih.gov/genbank/ (accession no. OM289665) (accessed on 15 November 2021).

## Supplementary Materials

The following are available online at https://www.mdpi.com/article/10.3390/ijms23031168/s1.
