# Supplementary material for: Deep Functional Profiling of Wild Animal Microbiomes Reveals Probiotic Bacillus pumilus Strains with a Common Biosynthetic Fingerprint
Source: Int J Mol Sci. 2022 Jan 21;23(3):1168. doi: 10.3390/ijms23031168 (PMC8835302; doi:10.3390/ijms23031168)
Supplement: Supplementary file 1 [file ijms-23-01168-s001.zip › ijms-1503320-supplementary.pdf]

International Journal of *Molecular Sciences*

MDPI

Supplementary Materials for

Deep functional profiling of wild animal microbiomes reveals probiotic *Bacillus pumilus* strains with a common biosynthetic fingerprint

Margarita N. Baranova[1], Arsen M. Kudzhaev[1], Yuliana A. Mokrushina[1,2], Vladislav V. Babenko[3], Maria A. Kornienko[3], Maja V. Malakhova[3], Victor G. Yudin[4], Maria P. Rubtsova[2], Arthur Zalevsky[1], Olga A. Belozerova[1], Sergey Kovalchuk[1], Yuriy N. Zhuravlev[4], Elena N. Ilina[3], Alexander G. Gabibov[1,2,*], Ivan V. Smirnov[1,2,*], Stanislav S. Terekhov[1,2,*]

1 Shemyakin-Ovchinnikov Institute of Bioorganic Chemistry of the Russian Academy of Sciences, 117997 Moscow, Russia

2 Department of Chemistry, Lomonosov Moscow State University, 119991 Moscow, Russia

3 Federal Research and Clinical Centre of Physical-Chemical Medicine of Federal Medical Biological Agency, Moscow 119435, Russia

4 Federal Scientific Center of the East Asia Terrestrial Biodiversity, Far-Eastern Branch of Russian Academy of Science, Vladivostok 690022, Russia.

* Correspondence should be addressed to gabibov@ibch.ru (A.G.G.); smirnov@ibch.ru (I.V.S.); sterekhoff@gmail.com (S.S.T.).

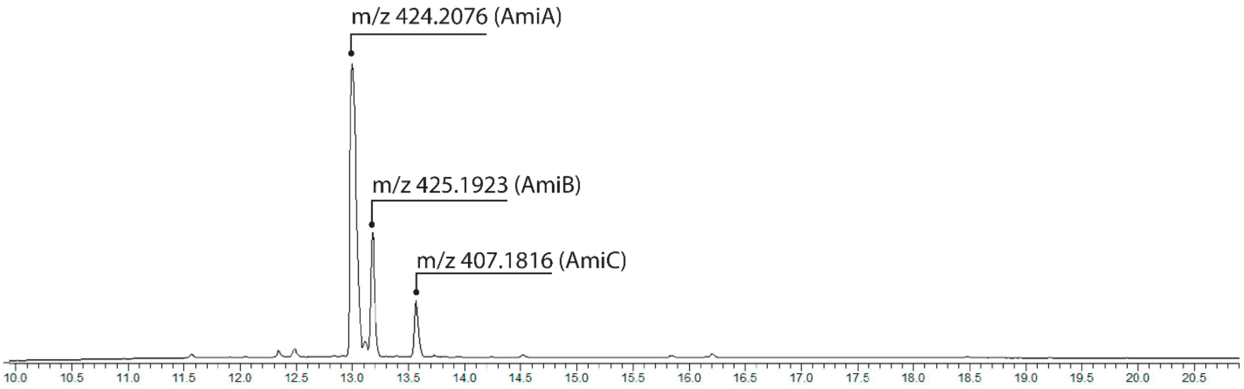

Figure S1. Representative LC-MS chromatogram of Ami fraction in *B. pumilus* culture medium. Ami (AmiA) and its degradation products are indicated. Amicoumacin B (AmiB) and amicoumacin C (AmiC) result from Ami lactonization (AmiC) followed by hydrolysis (AmiB).

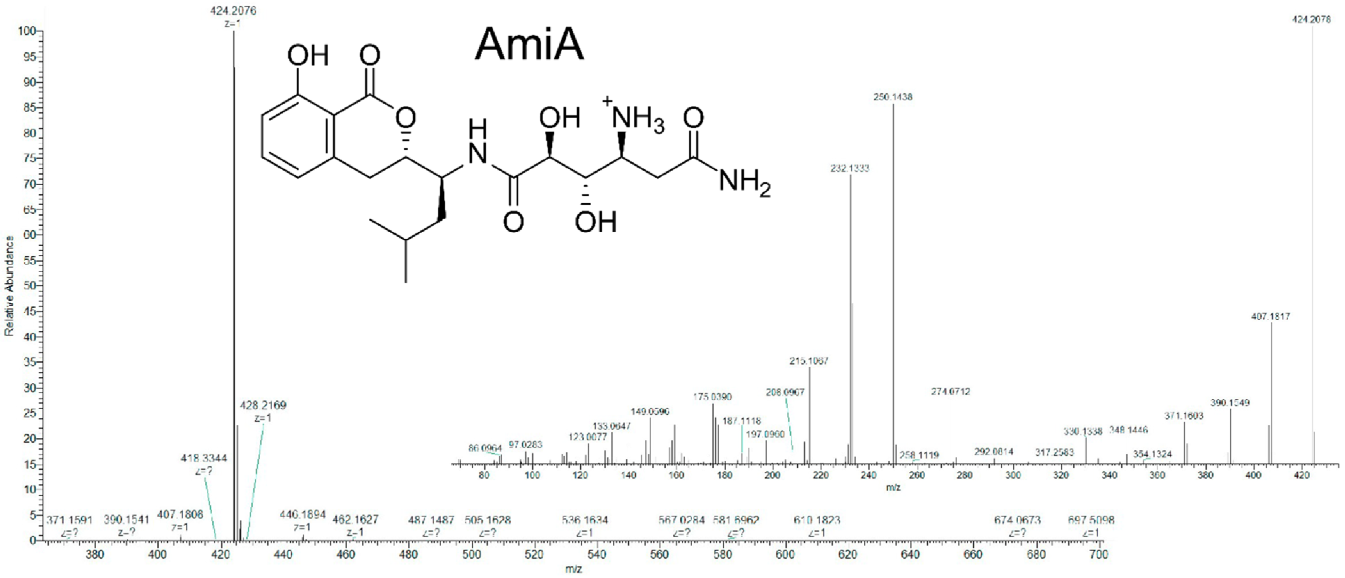

Figure S2. MS spectra of Ami (AmiA). AmiA $[M+H]^+$ m/z = 424.2076, Δ = 1 ppm. The positive mode HCD mass spectrum (inset).

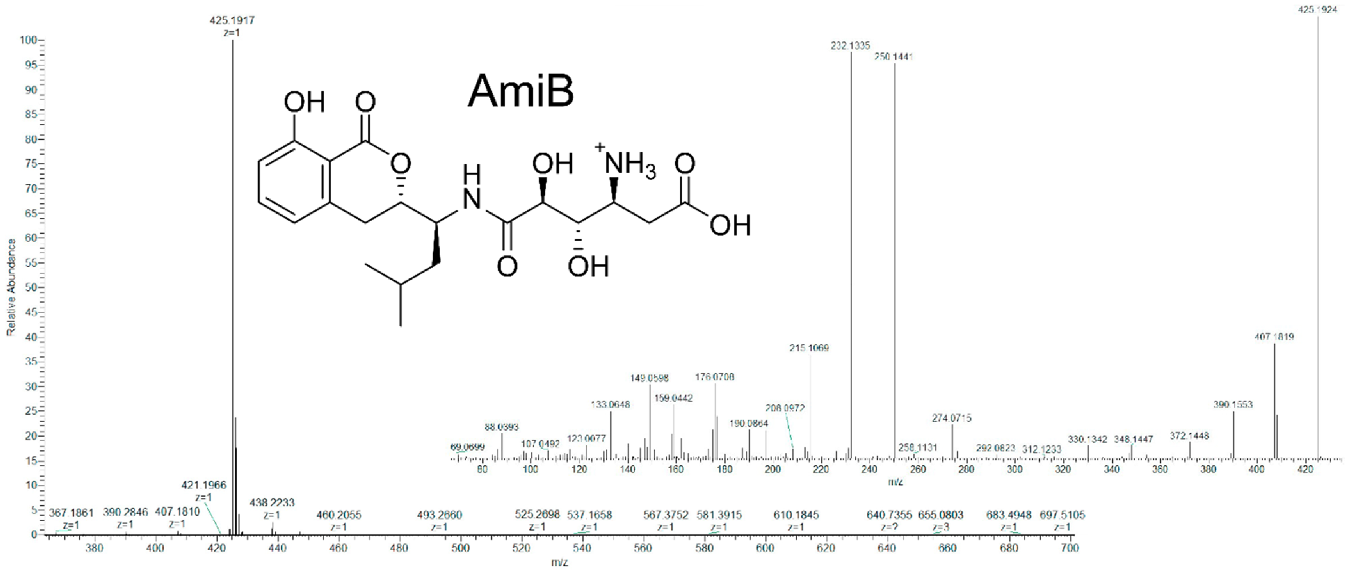

Figure S3. MS spectra of AmiB. AmiB $[M+H]^+$ m/z = 425.1917, Δ = 1 ppm. The positive mode HCD mass spectrum (inset).

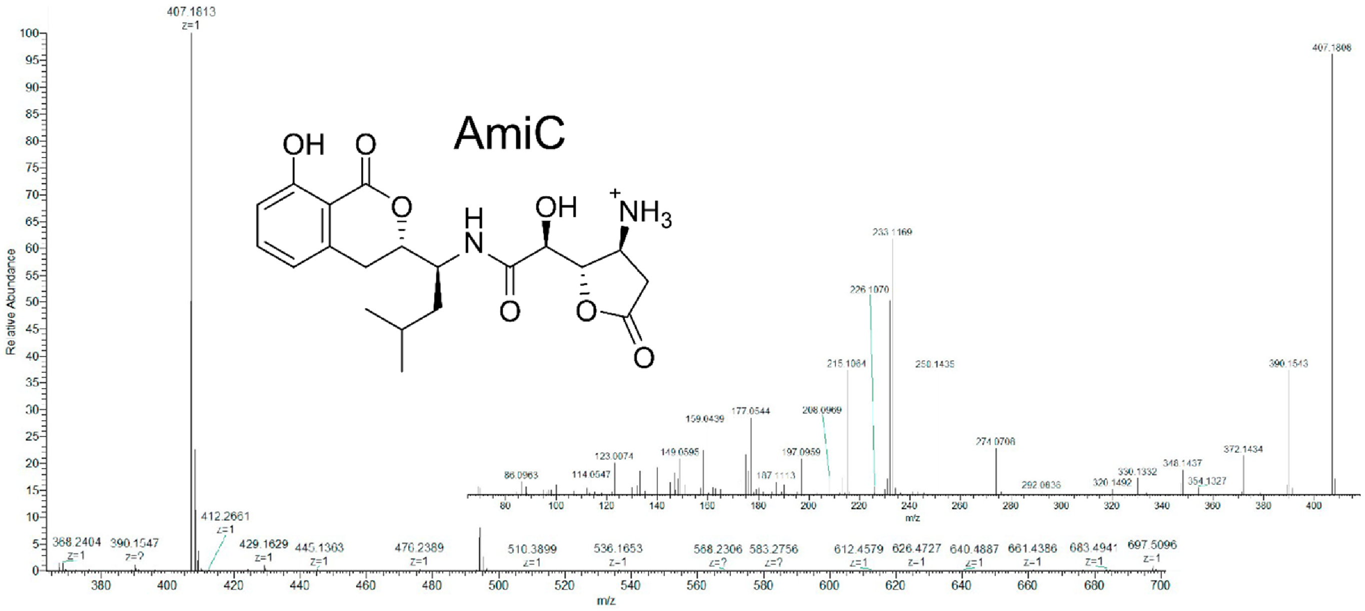

Figure S4. MS spectra of AmiC. AmiB $[M+H]^+$ m/z = 407.1813, Δ = 1 ppm. The positive mode HCD mass spectrum (inset).

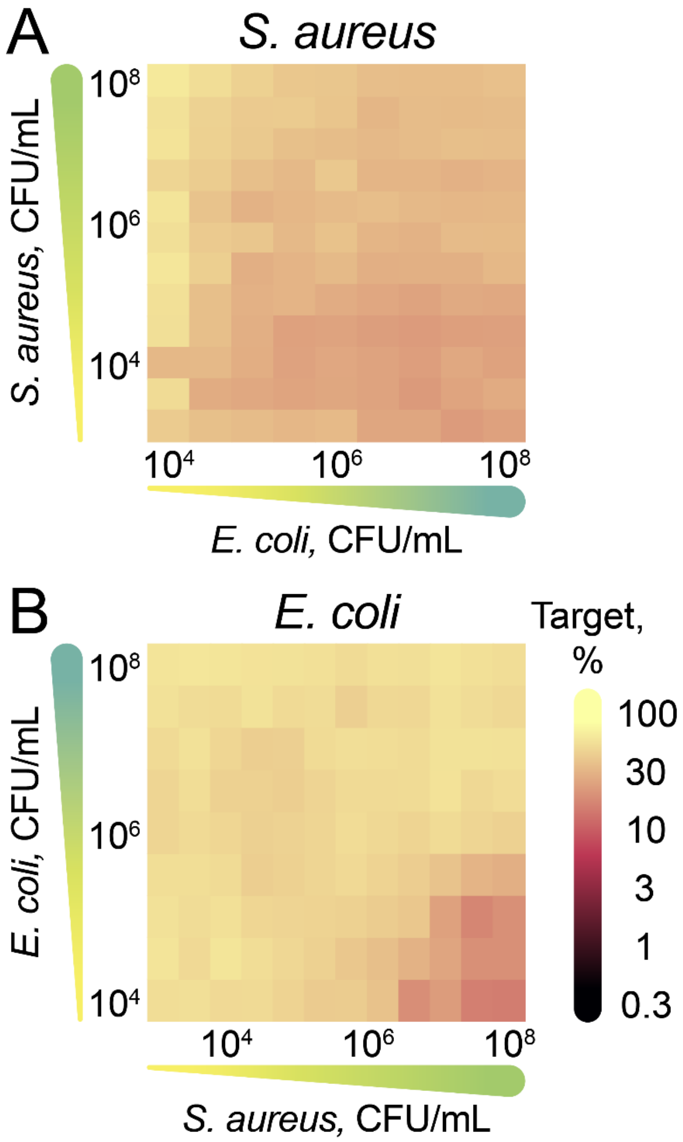

Figure S5. Cross-antagonistic activity landscapes of *S. aureus* and *E. coli*. *S. aureus* and *E. coli* were cocultivated using various cell ratios. Fluorescence of target *S. aureus* (**A**) or *E. coli* (**B**) cells was analyzed after 24 h of cocultivation. Heatmap indicates maximal proliferation of target *S. aureus* (**A**) or *E. coli* (**B**) cells estimated by relative fluorescence level.

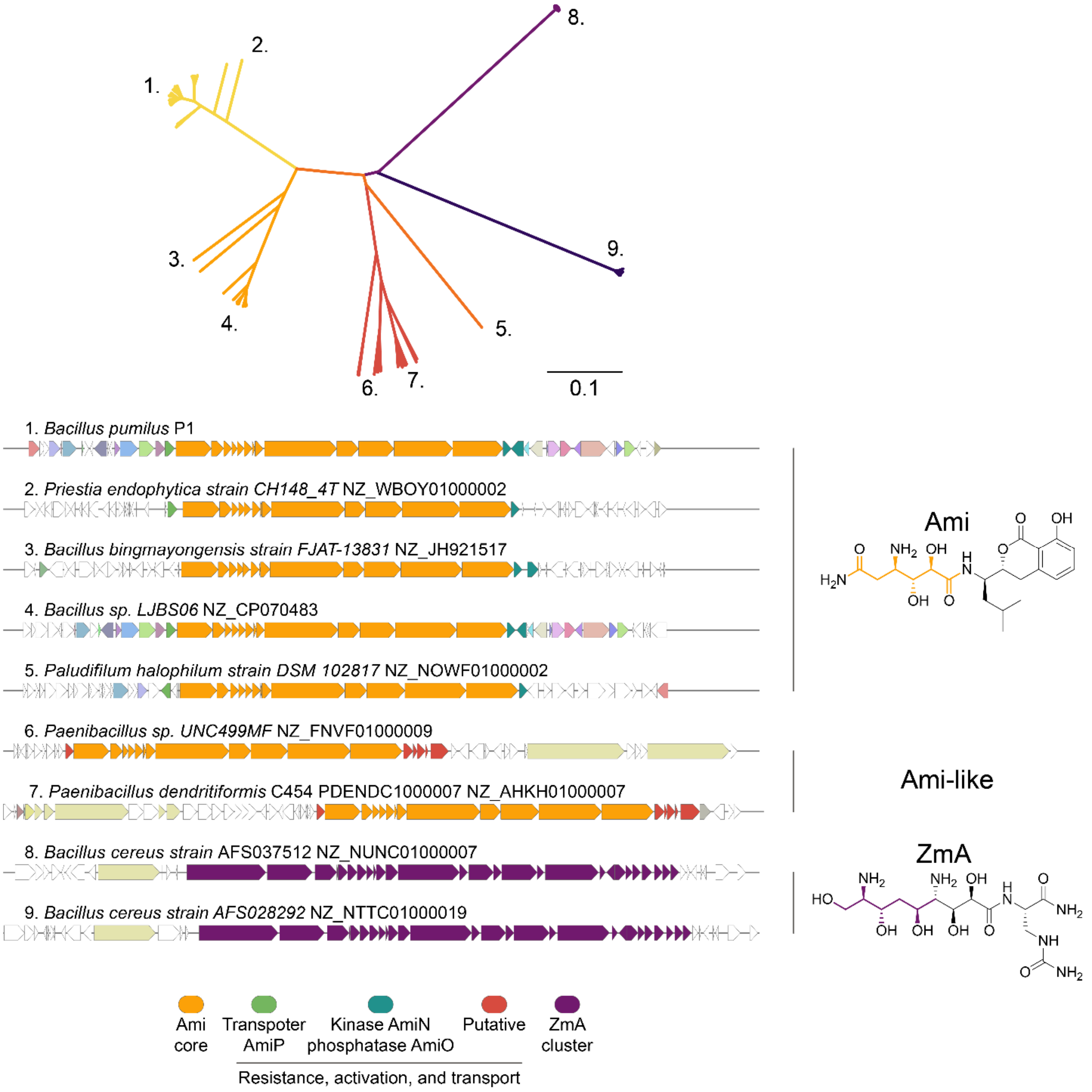

Figure S6. The architecture of Ami clusters (1-5), Ami-like clusters (6,7), and ZmA clusters (8,9). Ami clusters: (i) *B. pumilus* type (*B. pumilus* (**1**), *Priestia endophytica* (**2**)), (ii) *B. subtilis* type (*B. subtilis* (**4**), *B. bingmayongensis* (**3**)), *Paludifilum halophilum* (**5**), and (iii) *Paenibacillus* type (*P. dendritiformis* (**7**), *P. sp.* (**6**)). ZmA clusters (**8**,**9**). BGCs encoding Ami are colored with warm colors (yellow, orange, and red). BGCs encoding zwittermicin A (ZmA) are colored with cold colors (sapphire and violet). Core Ami enzymes are colored with orange. ZmA cluster is colored with violet. Transporter AmiP is colored with green. Kinase AmiN and phosphatase AmiO, mediate self-resistance toward Ami and Ami activation, respectively. AmiN and AmiO are colored with aquamarine. *Paenibacillus* genes encoding proteins putatively associated with transport/self-resistance are colored with red.
